# Inflammatory Bowel Disease Patients with a History of Cancer: Safety of Immunomodulators in a Multicenter Study

**DOI:** 10.3390/cancers17203293

**Published:** 2025-10-11

**Authors:** Roberto Mancone, Benedetto Neri, Clara De Francesco, Livio Bonacci, Mariasofia Fiorillo, Sara Concetta Schiavone, Anna Galbusera, Alba Sparacino, Anna Testa, Ambrogio Orlando, Emma Calabrese, Irene Marafini, Stefano Festa, Fabiana Castiglione, Walter Fries, Giovanni Monteleone, Livia Biancone

**Affiliations:** 1 Gastroenterology Unit, Department of Systems Medicine, University “Tor Vergata” of Rome, 00133 Rome, Italybenedettoneri@gmail.com (B.N.); irene.marafini@gmail.com (I.M.);; 2 Therapeutic GI Endoscopy Unit, Fondazione Policlinico Universitario Campus Bio-Medico, 00128 Rome, Italy; 3 IBD Unit, Department of Clinical and Experimental Medicine, University of Messina, 98122 Messina, Italy; 4 Gastroenterology Unit, Department of Clinical Medicine and Surgery, Università Federico II, 80138 Naples, Italyannatesta82@virgilio.it (A.T.);; 5 IBD Unit, “Villa Sofia-Cervello” Hospital, 90146 Palermo, Italy; 6 IBD Unit, Gastroenterology Department, San Filippo Neri Hospital, ASL Roma 1, 00135 Rome, Italy

**Keywords:** inflammatory bowel disease (IBD), new cancer, recurrent cancer, immunomodulators (IMM), biologics, clinical outcome

## Abstract

**Introduction**: The risk of new or recurrent cancer in inflammatory bowel disease (IBD) patients with a history of cancer treated with immunomodulators (IMMs), including conventional immunosuppressors (ISSs), biologics or small molecules is undefined. The primary aim was to assess the frequency of new or recurrent cancer in IBD patients treated with IMMs after first cancer. The secondary aim was to evaluate risk factors for new/recurrent cancer in the same IBD population. **Methods**: In a retrospective multicenter study, all IBD patients using any IMM after first (index) cancer were enrolled. Inclusion criteria: Crohn’s disease (CD) or ulcerative colitis (UC), history of any cancer, detailed clinical history, and follow-up after cancer of ≥6 months. Exclusion criteria: IMM use for ≤3 months. **Results**: In total, 122 IBD patients (84 CD, 38 UC) treated with IMMs after first cancer were enrolled (age 59.5 [26–89] years). Index cancer included (*n* = [%]) genitourinary tract cancer (18 [14.8]), non-melanotic skin cancer (NMSC) (17 [13.9]), breast cancer (15 [12.3]), thyroid cancer (13 [10.7]), melanoma (14 [11.4]), colorectal cancer (CRC) (11 [9.0]), hematopoietic cancer (9 [7.4]), prostatic cancer (8 [6.6]), neuroendocrine cancer (4 [3.3]), head and neck cancer (3 [2.5]), liver cancer (3 [2.5]), endometrium cancer (2 [1.6]), lung cancer (1 [0.8]) and others (3 [2.5]). ISSs after cancer included (*n* = [%]) thiopurines (10 [37]), methotrexate (MTX) (14 [51.9]) and others (3 [11.1]) Biologics included (*n* = [%]) TNF-inhibitors (36 [32.4]), vedolizumab (60 [53.6]), ustekinumab (45 [40.2]), small molecules (9 [7.3]) and others (6 [5.4]). In a median follow-up of 8 [1–45] years after index cancer, 12/122 (9.8%) patients using IMMs after cancer developed new or recurrent cancer. No risk factors for new/recurrent cancer (i.e., age at diagnosis of cancer, smoke, gender, IBD type, IMM use, duration before or after cancer) were identified. **Conclusions**: In a multicenter study, ISSs or biologics after cancer were not identified as risk factors for new or recurrent cancer in IBD. However, IMMs were used after a long-term interval from index cancer.

## Simple Summary

The risk of new or recurrent cancer in patients with Inflammatory Bowel Disease (IBD) with a history of malignancy treatet with immunomodulators is currently undefined. In a multicentre, retrospective study a total of 122 IBD patients were enrolled. Malignancies included cancers at low (*n* = 43), intermediate (*n* = 41) and high high risk (*n* = 38). In a median follow-up of 8 [1–45] years after index cancer, 12/122 (9.8%) patients using IMMs after cancer developed new or recurrent cancer. No risk factors for new/recurrent cancer were identified. Present data suggest that immunomodulators after cancer may be used, even though caution is required.

## 1. Introduction

Crohn’s disease (CD) and ulcerative colitis (UC) are inflammatory bowel disease (IBD) characterized by chronic intestinal inflammation and recurrent symptoms [1,2,3]. Although the etiology of IBD remains unknown, growing evidence suggest the role of an inappropriate mucosal immune response towards luminal antigens in the pathogenesis [1,2]. According to this concept, several immunomodulatory (IMM) treatments including conventional immunosuppressors (ISSs), biologics and small molecules, have been developed in IBD. IMM treatments currently aim to induce not only clinical but also endoscopic and histological remission. The achievement of these last two targets has been associated with a longer clinical remission [4]. Among conventional ISSs (thiopurines, methotrexate), azathioprine has been used since the 1970s in IBD [5]. More recently, new treatments specifically targeting or modulating the gut immune response have successfully been developed in IBD [6,7].

The worldwide use of IMMs initially raised concern regarding the potential cancer risk associated with their use [8,9]. In the general European population, the overall cancer risk not related to IMMs use ranges from 25% to 32% [10]. In IBD, a slightly increased overall cancer risk has been reported [11,12,13,14,15]. A higher risk has been reported for non-Hodgkin lymphoma (NHL) in CD and for skin cancers, particularly non-melanoma skin cancer (NMSC) in both CD and UC [11,12,13,14,15]. Colorectal cancer (CRC) risk is also increased in IBD-related colitis, occurring at a younger age than in the general non-IBD population [16].

A huge amount of data addressing the risk of first cancer using IMM in IBD patients is currently available, supporting the safety of these compounds. A slightly increased risk of NMSC, melanoma and lymphoma [17] has, however, been associated with ISS and TNFα-inhibitor (TNFi) use, particularly in CD [17].

Different from first cancer, the risk of new or recurrent cancer in IBD patients treated with IMMs after a prior malignancy is still undefined. Limited evidence regarding the use of ISSs and TNFis after cancer suggests no increased risk of new or recurrent cancer, reported in about 27% of IBD patients [18,19,20]. Fewer data are available regarding the more recent use of biologics other than TNFis and of small molecules in IBD patients with prior cancer. Moreover, CD and UC patients with a current or prior malignancy are excluded from RCTs, thus, further limiting evidence in this regard. Importantly, current data regarding this topic is mostly derived from studies including IBD patients treated with ISS or biologics ≥ 5 years after cancer remission or with a history of cancer at low recurrence risk.

Worldwide cancer survival significantly increased during the last decades in the general population, and cancer survivors show a higher risk of new cancer (RR 1.4) [21]. The issue regarding cancer risk from using IMMs after cancer will therefore involve a higher number of IBD patients in the next future. IMM use in patients with a history of cancer therefore represents a challenge in the clinical management of patients with IBD.

On the basis of these observations, the primary aim of our study was to assess the frequency of new or recurrent cancer in IBD patients treated with ISSs, biologics or Janus Kinases-inhibitors (JAKis) after a diagnosis of cancer. The secondary aim was to assess clinical characteristics of IBD patients developing new or recurrent cancer after IMM use and to search for related risk factors.

## 2. Materials and Methods

### 2.1. Study Protocol

In a retrospective, multicentre study, all IBD patients with a previous diagnosis of cancer treated with ISSs, biologics or small molecules were enrolled. IBD was diagnosed and classified according to standard criteria [1,2,3]. For each patient, demographic and clinical characteristics together with histological reports of cancer were previously prospectively reported in clinical records at the time of scheduled visits.

For the purpose of the study, IBD patients fulfilling the inclusion criteria were enrolled in 5 tertiary referral Italian IBD centers: University “Tor Vergata” (Rome), University of Messina (Messina), University Federico II (Naples), “Villa Sofia-Cervello” Hospital (Palermo) and “S. Filippo Neri” Hospital (Rome).

### 2.2. Study Population

In each IBD center, patients were enrolled according to the following inclusion criteria: (1) diagnosis of CD or UC according to current guidelines [22]; (2) IBD patients referring to the above reported IBD units; (3) concomitant diagnosis of IBD and cancer; (4) well defined diagnosis of cancer including histological report; (5) diagnosis of cancer either before or after the diagnosis of IBD; (6) ISS, biologics or small molecule use at any time and for any indication after index cancer and (7) clinical follow-up after the diagnosis of cancer for ≥4 months. Exclusion criteria were (1) ISSs, biologics or small molecules used for ≤3 months and (2) incomplete data in clinical records.

In each IBD center, demographic and clinical characteristics of each patient were already prospectively reported in a database including age, gender and age at time of diagnosis of cancer and of IBD; histotype of index; new or recurrent cancer; cancer stage and treatment (chemotherapy, radiotherapy, immunotherapy, hormonal therapy); cancer survival (yes/no); time interval from the diagnosis of index cancer to IMM use; follow-up duration after index cancer (months); smoking status (yes vs. no/ex); IBD duration (years); UC extent (proctitis, E1; left-sided, E2; extensive, E3) [22]; CD site (ileum, L1; colon, L2; ileum-colon, L3; upper GI, L4); CD behavior (non-stricturing–non-penetrating, B1; fibrostricturing, B2; penetrating, B3) [22]; perianal disease (yes/no); treatment with ISSs, biologics or small molecules (yes/no, type, duration) before or after index cancer and other treatments for IBD or for other conditions (yes/no, type).

For each patient, the outcome was recorded in terms of (1) the development of new or recurrent cancer after treatment with ISS, biologics or small molecules; (2) the length of follow-up after IMM treatment and (3) cancer-related death (yes/no). For the purpose of the study, the frequency of new/recurrent cancer was compared between IBD patients, stratified according to prior cancer at high risk, intermediate risk or low risk of recurrence [23,24].

The study protocol was approved by the Territorial Independent Ethics Committee of “Lazio Area 2”, Italy (protocol n. 94.24 CET2). Patient consent was waived as data were retrospectively collected; the investigation did not add risk for participants and all data were de-identified.

### 2.3. Statistical Analysis

Data were expressed as median [range]. The normal distribution of parametric continuous variables was assessed by using the Kolmogorov–Smirnov test and defined by a *p*-value < 0.05. Differences between qualitative and quantitative variables were assessed by the Pearson χ^2^ test, Student’s *t*-test or Mann–Whitney U-test, as appropriate. Univariate and multivariate logistic regression models were used for assessing risk factors for new/recurrent cancer (odds ratio, OR [95% CI]). Variables considered as potential risk factors for new/recurrent cancer included gender, smoking status, IBD type, age, type and duration of IMM treatment before and after index cancer and cancer treatment (surgery, chemotherapy, radiotherapy, hormonal therapy).

Statistical significance was considered for all variables in the case of *p*-value < 0.05. Statistical analysis was performed with IBM-SPSS statistical software ver. 26.0.

## 3. Results

### 3.1. Study Population

The study population included 122 IBD patients treated with ISSs, biologics and/or small molecules after the diagnosis of cancer. Demographic and clinical characteristics of IBD patients are summarized in Table 1. As shown, among the 122 IBD patients enrolled (58 [47.5%] females), there were 84 (68.9%) patients with CD and 38 (31.1%) with UC. The median age of IBD patients was 59.5 [26–89] years, with a median IBD duration of 13.5 [1–59] years. The CD site included ileum (L1) in 50 (59.5%), colon (L2) in 4 (4.8%), ileum-colon (L3) in 30 (35.7%) and upper GI (L4) in 7 (8.3%) patients. CD behavior was non-stricturing–non-penetrating in 24 (B1) (28.5%), fibrostricturing (B2) in 45 (53.6%) and penetrating (B3) in 15 (17.9%) patients. Perianal disease was observed in 17 (20.2%) patients. In UC, the disease extent included proctitis (E1) in 2 (5.3%), left-sided colitis (E2) in 13 (34.2%) and extensive colitis (E3) in 23 (60.5%) patients. At enrollment, 44 (36.1%) IBD patients were active smokers. Conventional therapies used for IBD before the diagnosis of cancer included 5-aminosalicylates in 97 (79.5%), sulfasalazine in 21 (17.2%), systemic corticosteroids in 84 (68.9%) and low-absorbable corticosteroids in 67 (55%) patients.

### 3.2. Immunomodulatory Treatments Before Index Cancer

Table 2 summarizes IMM treatments used before index cancer in IBD patients and, separately, in patients with CD and UC. Among the 122 IBD patients, 41 (33.6%) were exposed to ISSs, 46 (37.7%) to biologics and 1 (0.8%) to JAKi before index cancer. In particular, ISSs before index cancer included thiopurines in 34 (82.9%), methotrexate in 6 (14.6%) and other ISSs in 3 (7.3%) patients. Among these three patients, one UC patient was treated for 36 months with mycophenolate mofetil before the diagnosis of renal oncocytoma, due to a membranous glomerulonephritis. The second UC patient was treated for 96 months with combined tacrolimus and everolimus before the occurrence of NMSC. Finally, one CD patient was treated for 96 months with everolimus before the occurrence of urinary tract carcinoma (in situ). In these last two patients, the indication for ISSs was liver transplantation. The median duration of ISS treatment in these 41 IBD patients was 38 [3–228], 36 [3–89] and 66 [36–96] months for thiopurines, methotrexate and other ISSs, respectively (Table 2).

Before index cancer, biologics were used in 46 (37.7%) patients and JAKi in 1 (0.8%) patient (Table 2). Biologics used before cancer in these 46 IBD patients included TNFis in 40 (88.8%), vedolizumab in 10 (25%) and ustekinumab in 4 (10%) patients. The median duration of biologic or small molecules treatment before index cancer was 36 [3–180], 10.5 [3–63] and 42.5 [1–58] months, respectively (Table 2). One UC patient was treated with tofacitinib for 18 months before the first cancer.

### 3.3. Index Cancer: Characterization

Characteristics of index cancer occurring in the 122 IBD patients treated with IMMs after the diagnosis of cancer are summarized in Table 3. As shown, cancer before IMM use in the 122 IBD patients involved (Table 3) the skin in 31 (25.4%), genitourinary tract in 18 (14.8%), prostate in 8 (6.6%), breast in 15 (12.3%), thyroid in 13 (10.7%), colon in 11 (9.0%), hematopoietic system in 9 (7.4%), neuroendocrine system in 4 (3.3%), head–neck district in 3 (2.5%), liver in 3 (2.5%), endometrium in 2 (1.6%), lung in 1 (0.8%) and other tissues in 4 (3.3) patients. The 31 skin cancers included 17 NMSCs (13.9%) and 14 (11.4%) melanomas.

In the tested IBD population, index cancer occurred at a median age of 50.5 [2–79] years.

### 3.4. Index Cancer: Treatment

In the 122 patients, the median follow-up duration after the diagnosis of index cancer was 8 [1–45] years. Cancer treatment included surgery in 108 patients (88.5%, achieving R0 in 106 [98.1%]), chemotherapy in 30 (24.6%), hormonal therapy in 53 (43.4%) and/or radiotherapy in 19 (15.6%) patients. Treatment responsiveness was complete in 120 (98.3%) patients before IMMs. Responsiveness was defined as no cancer visualized by imaging during the follow-up period. One CD patient showed no responsiveness to oncologic treatment (surgery and chemotherapy) and developed metastatic prostatic adenocarcinoma while using vedolizumab. Partial response to thalidomide was observed in one CD patient with smoldering multiple myeloma treated with vedolizumab and ustekinumab.

### 3.5. Immunomodulatory Treatments After Index Cancer

Table 4 summarizes IMM treatments used after index cancer in IBD patients and, separately, in patients with CD and UC. In the tested population of 122 IBD patients, ISSs were used after cancer in 27 (22.1%) patients, including thiopurines in 10 (37%) and methotrexate in 14 (51.9%) patients. Other ISS treatments were used for not IBD in 3 (11.1%) additional patients (Table 4). The median duration of ISS treatment after index cancer was 29 [4–200] months (Table 4).

Biologics were used after index cancer in 113 (92.6%) patients (Table 4). Among these 113 IBD patients using biologics, 36 (31.8%) were treated with TNFis, 60 (53.1%) with vedolizumab, 45 (39.8%) with ustekinumab, 5 (4.4%) with risankizumab and 1 (0.8%) with secukinumab. The median duration (months) of biologic treatments after index cancer was 38 [3–180] for TNFis, 24 [3–84] for vedolizumab, 24 [3–66] for ustekinumab, 6 [3–12] for risankizumab and 48 [48–48] for secukinumab (Table 4).

Small molecules were used after index cancer in nine (7.4%) IBD patients (Table 4). These treatments included tofacitinib in three (33.3%) and upadacitinib in four (44.4%) patients, filgotinib in one and ozanimod in one (11.1%) patient each. The median duration of small molecule treatment was 17 [3–18], 6 [5–6], 12 [12–12] and 4 [4–4] months, respectively.

In the group of 122 IBD patents, the median time interval from index cancer to IMM use was 27 [0–312] months for ISSs, 48 [1–432] months for biologics and 108 [12–396] months for small molecules.

### 3.6. New or Recurrent Cancer in Patients Treated with Immunomodulators After Cancer

Overall, among the 122 IBD patients treated with any IMM after index cancer, a new or recurrent cancer occurred in 12 (9.8%) patients in a median follow-up of 8 [1–45] years.

**Figure 1:**
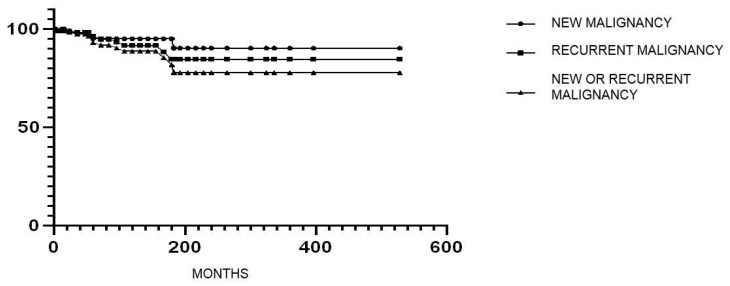
The figure shows the Kaplan–Meier survival analysis for new and/or recurrent cancer in IBD patients treated with IMMs after a prior cancer. Findings from 3 IBD groups are reported: (1) all IBD patients with either new or recurrent cancer; (2) IBD patients with new cancer and (3) IBD patients with recurrent cancer.

The incidence of new/recurrent cancer in the tested IBD population was 1.05 per 100 person-years. Among these 122 IBD patients, 5 (4.1%) developed cancer recurrence and 7 (5.7%) a new cancer. In particular, one patient developed both cancer recurrence and new cancer, while two patients developed two new cancers (Table 5). Cancer recurrence included thyroid (*n* = 2), prostate (*n* = 2) and CRC (*n* = 1). Differently, new cancers included NMSC (*n* = 7), melanoma (*n* = 2) and salivary gland carcinoma (*n* = 1). Details regarding index cancer, recurrent or new cancer and type/duration of IMM treatments after cancer are summarized in Table 5. The median time interval from index cancer to a new or recurrent cancer was 60 [3–183] months. The median time interval from IMM use to the diagnosis of new or recurrent cancer was 38.5 [3–108] months. Kaplan–Meier survival analysis for a new and/or recurrent cancer in IBD patients treated with IMMs after a prior cancer is reported in Figure 1.

Patients with or without new/recurrent cancer were comparable in terms of the frequency of biologic/JAKi use and duration/type of IMM treatments before and after index cancer (Table 6 and Table 7).

No difference in terms of the frequency of new/recurrent cancer was observed when comparing patients stratified according to prior cancer at low, intermediate or high risk (index cancer at low vs. intermediate risk: 4/43 [9.3%] vs. 3/41 [7.3%]; *p* = 0.94; low vs. high risk: 4/43 [9.3%] vs. 5/38 [13.2%]; *p* = 0,84: intermediate vs. high risk: 3/41 [7.3%] vs. 5/38 [13.2%]; *p* = 0.62).

The family history of any cancer was recorded in 23/122 (18%) IBD patients. The frequency of new/recurrent cancer did not significantly differ between patients with or without family history of any cancer (5/12 [41.7%] vs. 18/110 [16.3%]; *p* = 0.08).

At univariate analysis, no risk factors for new or recurrent cancer were observed among the variables considered (Table 8).

## 4. Discussion

Immunomodulatory treatments currently represent the most effective long-term treatments in IBD. The relatively recent worldwide use of these therapeutic strategies in IBD gave rise to concerns regarding their safe use in patients with a history of cancer. Evidence currently support that the overall risk of first cancer is not increased by their use [25]. A slightly higher risk of lymphoma and skin cancer has, however, been reported using thiopurines and/or TNFis in IBD [16]. Differently, evidence regarding the risk of developing a new or recurrent cancer using any IMM in IBD patients with a history of cancer is currently limited. In 2014, a CESAME study reported that thiopurine use was not associated with an increased risk of new or recurrent cancer in IBD patients with a prior malignancy [20]. A multicenter retrospective study involving 333 IBD patients with a concomitant history of cancer reported that using TNFis or ISSs was not associated with an increased risk of incident cancer [19]. However, due to the relevance of this topic and to the relatively few available evidence in this regard, no conclusive statements can be made [25]. In particular, TNFis were reported to be potentially used in IBD patients with previous cancer, but the decision should be discussed with oncologists [25]. According to preliminary evidence, ustekinumab and vedolizumab were reported not to increase the risk of new or recurrent cancer, while no recommendations were given regarding JAKi [25]. More recently, an interim analysis of data prospectively collected from the American SAPPHIRE registry, reported a non-statistically significant association between IMM use and new/recurrent cancer in IBD patients with a history of neoplasia [26]. However, most of the data regarding the risk of new or recurrent cancer in IBD patients using IMM after a diagnosis of cancer are derived from observational studies, often including limited populations with a short follow-up. Moreover, in most of the studies, IMMs were given >5 years after index cancer, mostly characterized by a low recurrence risk. Overall, available studies, in this regard, conclude that there is insufficient evidence to make recommendations. On the basis of these observations, whether IMM may be safely used in IBD patients with a history of cancer needs to be further investigated.

At this purpose, in the present retrospective multicenter study, IBD patients were enrolled in five referral centers. Accordingly, the study population was mostly represented by patients with severe IBD. Pancolitis was indeed observed in more than 60% of UC patients, CD phenotype was mostly fibrostricturing and a long median IBD duration was reported. This observation may account for clinical indication to use IMMs despite first cancer.

Index cancer was represented by skin cancer in more than one fourth of patients, including melanoma in more than one tenth of the study population. Skin cancer represented the most frequent index cancer in the tested IBD population, in agreement with current evidence [12,13,14]. This finding also suggests that additional efforts should be made by IBD-dedicated gastroenterologists in terms of dermatological screening and prevention. This is particularly true for patients using thiopurines and/or TNFis [12,13,14]. According to current European guidelines [25], no standardized dermatological surveillance protocols are available in IBD. However, for the general non-IBD population, surveillance was tailored according to patients’ risk, also considering IMM use. The distribution of the type of index cancer in our population was expected and in line with previous studies (skin cancer followed, in order, by genitourinary, breast, thyroid, CRC, hematopoietic cancer) [26].

Despite the expected high prevalence of NMSC, prior cancer was represented by aggressive cancers in quite a high proportion of patients. In these patients, IMMs were used by IBD-dedicated gastroenterologists according to current clinical criteria, after a multidisciplinary assessment with oncologists. In the tested IBD population, the median time interval from index cancer to IMM treatments was <3 years, thus shorter than the 5-years safety interval required for more aggressive cancers. However, the time interval from the diagnosis of cancer to biologic use was longer in patients at higher risk of cancer recurrence (i.e., breast cancer vs. NMSC: 60 vs. 6 months, *p* = 0.008). The same was observed for genitourinary malignancies versus NMSC (90 vs. 6 months, *p* = 0.01). This finding is mostly related to a different recurrence risk of these cancers. Potentially aggressive malignancies indeed bear, by definition, a higher recurrence risk, regardless of IMM treatments, as well known by the scientific community.

Cancer was diagnosed at early stage in quite all the enrolled patients. Therefore, treatment included surgery in most of the patients (108 out of 122), being curative in most of them (106 out of 108). However, among the 108 patients undergoing surgical treatment, 17 (15.7%) also received adjuvant radiotherapy, 21 (19.4%) neo and/or adjuvant chemotherapy and 9 (8.3%) both radio- and chemotherapy. These observations therefore support the persistence of a recurrence risk after surgery in these patients. The tested IBD population showed various types of malignancies diagnosed at early stages, characterized by a variable recurrence risk. Despite almost half of the tested IBD population showing aggressive cancer types, a low frequency of new or recurrent cancer (9.8%) was observed in a median follow-up of 8 years after index cancer.

New or recurrent cancer in a median follow-up of 4.8 years after the first cancer was reported in 36 out of 210 IBD patients (17%) included in a recent prospective multicenter SAPPHIRE register [26]. The cancers were mostly represented by NMSC (42%), melanoma (11%) or other solid neoplasms (33%) [26]. The incidence of new or recurrent cancer in IBD patients treated with IMMs after cancer did not significantly differ when comparing our tested IBD population with the IBD cohort from the SAPPHIRE registry (12/122 [9.8%] vs. 36/210 [17%], *p* = 0.07) [26]. Accordingly, no significant difference was observed in terms of the rate of new/recurrent cancer when comparing our IBD population using IMMs after cancer with the same cohort of IBD patients with a prior cancer but not using IMMs (12/122 [9.8%] vs. 10/95 [11%]; *p* = 0.87) [26]. The observed frequency of new/recurrent cancer using IMMs is in agreement with current evidence [25]. Heterogeneity in terms of risk factors for cancer recurrence and of characteristics of the study populations (i.e., age, family and personal history of cancer) may, however, give rise to discrepant findings.

In the tested population, TNFi duration after cancer was at the limit of the statistical significance at univariate analysis (OR 3.01, *p* = 0.09). TNFi duration after index cancer was comparable between patients with versus without new/recurrent cancer (*p* = 0.17), thus supporting that TNFi duration did not represent a significant risk factor in our population, in agreement with current evidence at this regard [25].

Findings from the present IBD population therefore support the safety of IMM treatments in patients with a history of cancer. This observation is relative to a multicenter study including patients recruited and assessed in tertiary IBD centers by dedicated gastroenterologists. This suggests an appropriate selection of the subgroups of patients deserving IMM treatments after a diagnosis of cancer. Nevertheless, present findings do not allow us to state practical framework regarding waiting times before IMM use in IBD based on cancer type and stage. No conclusive statements in this regard are indeed reported in current European guidelines [25], due to heterogeneity of cancer types/stage, IBD populations and prior IMM use in different studies. Indications for IMM treatments in IBD therefore need a multidisciplinary approach including gastroenterologists and oncologists, followed by a decision shared with the patient. An accurate stratification of IBD patients in relation to the risk of new/recurrent cancer related to the type and stage of index cancer may lead to a proper assessment of the benefit/risk balance when choosing IMMs in these patients.

Among the main limitations of the present study, there is the retrospective design and the inclusion of all IBD patients with a history of any cancer type and stage. The oncological outcome was therefore assessed in patients with previous cancers bearing a different recurrence risk. Moreover, due to the study design, the quite long median follow-up of patients from the diagnosis of index cancer (8 years), showed wide interindividual variations (range 1–45 years). The small sample size for JAK inhibitors and newer biologics (ustekinumab, vedolizumab, risankizumab) is an additional limitation. This finding is conceivable due to the recent use of these IMMs in IBD, thus limiting the number of treated patients, particularly with a history of cancer. Nevertheless, present findings may add data to the few evidence in this regard. Although the retrospective design of the study is the main limitation of our study, selection bias was minimized across centers by sharing well defined inclusion and exclusion criteria. In each center, all IBD patients treated with IMMs for ≥3 months after a diagnosis of any cancer and subsequently followed up for ≥4 months were indeed considered in the analysis.

Among the strengths of the study, the multicenter study design including five referral IBD centers assessing quite a large number of patients classified according to standard criteria [22]. The multicenter nature of the study should also allow an objective evaluation of the risk of new/recurrent cancer using IMMs in the tested IBD population. Moreover, data from each patient were prospectively recorded in clinical records during scheduled follow-up visits. Patients showed demographic and clinical characteristics comparable to the general IBD population, thus avoiding bias related to inappropriate subgroups selection. Whether using IMMs may increase the risk of new or recurrent cancer in IBD patients with a previous malignancy is still undefined. Present findings may therefore add new information regarding the recurrence risk using IMMs, including several types of biologics, in IBD patients with a history of cancer. Considering the few available evidence at this regard, the reported observations provide additional “real world” data, which may useful clinical management of IBD patients with a history of malignancy.

## 5. Conclusions

In conclusion, findings from our retrospective multicenter study suggest that in the tested population the use of ISSs, biologics and small molecules was not associated with an increased risk of new or recurrent malignancy in IBD patients with a history of cancer. Despite concerns regarding the immunomodulatory effects of these treatments, our data support their safety even in patients with a history of cancer, provided that there is a careful selection of patients and an appropriate risk stratification. The observed low frequency of new or recurrent cancer in the tested cohort, although limited by the retrospective nature of the study, adds support to the suggested safety of IMM in these patients. However, it is important to note that the study population predominantly consisted of IBD patients with stages of malignancies at low recurrence risk. Moreover, the timing for IMM use after cancer was carefully assessed in a multidisciplinary approach with oncologists. In the tested IBD population, no significant risk factors for new or recurrent cancer emerged in patients treated with IMM after cancer. However, larger prospective studies including patients with longer follow-up are needed in order to provide evidence for proper management of IBD patients with a history of cancer.

The need for a multidisciplinary approach involving both oncologists and gastroenterologists is clearly highlighted by our findings, as timely and personalized therapy management is crucial for ensuring the safety and efficacy of immunomodulatory treatments. Further research is essential in order to consolidate our understanding regarding the safety profile of IMM and to define the effects of these treatments in IBD patients with a prior cancer at high risk of recurrence.

## Figures and Tables

**Table 1:** Demographic and clinical characteristics of IBD patients treated with immunomodulators (IMMs) after the occurrence of cancer: difference between patients with versus without new or recurrent cancer.

	Study Population (*n* = 122)	New/Recurrent Cancer (*n* = 12)	No new/Recurrent Cancer (*n* = 110)	*p*
**Age, median [range]**	59.5 [26–89]	59 [27–86]	60 [26–89]	0.42
**Age at diagnosis of cancer, median [range]**	50.5 [2–79]	48 [17–79]	51 [2–75]	0.57
**Gender (F), *n* (%)**	58 (47.5)	4 (33.3)	54 (49.1)	0.29
**IBD duration, median [range]**	13.5 [1–59]	12 [8–45]	16 [1–59]	0.59
**Time interval from first to second cancer, median [range]**	60 [3–183]	60 [3–183]	n.a.	n.a.
**Time between ISSs/biologics and** **second cancer, median [range]**	38.5 [3–108]	38.5 [3–108]	n.a.	n.a.
**Follow-up after cancer, median [range]**	8 [1–45]	9 [3–15]	7 [1–45]	0.95
**Death, *n* (%)**	0 (0)	0 (0)	0 (0)	n.a.
Active smoking (yes at enrollment)	44 (36.1)	3 (25)	41 (37.3)	0.6
Extraintestinal manifestations, *n* (%)	24 (19.7)	1 (8.3)	23 (20.9)	0.51
**Age at diagnosis of IBD**				
≤16 years	6/122 (4.9)	1/12 (8.3)	5/110 (4.5)	0.89
>17 and ≤40 years	58/122 (47.5)	6/12 (50)	52/110 (47.3)	0.95
40 years	58/122 (47.5)	5/12 (41.7)	53/110 (48.2)	0.9
**UC**	38 (31.1)	4 (33.3)	34 (30.9)	1.0
E1	2/38 (5.3)	0/4 (0)	2/34 (5.8)	n.a.
E2	13/38 (34.2)	2/4 (50)	11/34 (32.4)	0.83
E3	23/38 (60.5)	2/4 (50)	21/34 (61.8)	0.85
**CD**	84 (68.9)	8 (66.7)	76 (69.1)	0.87
L1	50/84 (59.5)	2/8 (25)	48/76 (63.2)	0.13
L2	4/84 (4.8)	1/8 (12.5)	3/76 (3.9)	0.85
L3	30/84 (35.7)	5/8 (62.5)	25/76 (32.9)	0.27
L4	7/84 (8.3)	1/8 (12.5)	6/76 (7.9)	0.81
B1	24/84 (28.6)	2/8 (25)	22/76 (28.9)	0.91
B2	45/84 (53.6)	5/8 (62.5)	40/76 (52.6)	0.96
B3	15/84 (17.8)	1/8 (12.5)	14/76 (18.5)	0.98
**Perianal Disease, *n* (%)**	17/84 (20.2)	2/8 (25)	15/76 (19.7)	0.88

Abbreviations: IBD = inflammatory bowel disease; CD = Crohn’s disease; UC = ulcerative colitis; ISSs = conventional immunosuppressors; n.a. = not applicable.

**Table 2:** Immunosuppressors, biologics and small molecules before index cancer.

	Immunosuppressors			Biologics			Small Molecules
IBD Patients, *n*=	41			46			1
	Thiopurines (*n* = 35)	Methotrexate (*n* = 5)	Others (*n* = 3)	TNFi (*n* = 40)	Vedolizumab (*n* = 10)	Ustekinumab (*n* = 4)	Tofacitinib (*n* = 1)
Duration, median [range], months	38 [3–228]	36 [3–89]	66 [36–96]	36 [3–180]	10.5 [3–63]	42.5 [1–58]	18 [18]
	**Immunosuppressors**			**Biologics**			**Small Molecules**
**CD patients, *n*=**	**32**			**34**			**0**
	**Thiopurines** **(*n* = 28)**	**Methotrexate** **(*n* = 5)**	**Others** **(*n* = 1)**	**TNFi** **(*n* = 30)**	**Vedolizumab** **(*n* = 5)**	**Ustekinumab** **(*n* = 4)**	**Tofacitinib** **(*n* = 0)**
Duration, median [range] months	38.5 [3–228]	36 [3–89]	96 [96–96]	32 [30–180]	9 [3–63]	21.5 [1–48]	n.a.
	**Immunosuppressors**			**Biologics**			**Small Molecules**
**UC patients, *n*=**	**9**			**12**			**1**
	**Thiopurines** **(*n* = 7)**	**Methotrexate** **(*n* = 0)**	**Others** **(*n* = 2)**	**TNFi** **(*n* = 10)**	**Vedolizumab** **(*n* = 0)**	**Ustekinumab** **(*n* = 5)**	**Tofacitinib** **(*n* = 1)**
Duration, median [range] months	35 [3–72]	n.a.	66 [36–96]	24 [8–108]	n.a.	14 [3–24]	18 [18–18]

Abbreviations: JAKi = Janus-kinase inhibitor; TNFi = tumor necrosis factor-α inhibitor; CD = Crohn’s disease: UC = ulcerative colitis; n.a. = not applicable.

**Table 3:** Index cancer: site and characteristics in IBD patients and in patients with CD versus UC.

Index Cancer	IBD (*n* = 122)	CD (*n* = 84)	UC (*n* = 38)	*p*
**Skin cancer, *n* (%)**	31 (25.4)	21 (25)	10 (26.3)	0.87
***Non-melanoma skin cancer, n (%)***	17 (13.9)	12 (14.3)	5 (13.2)	0.87
Basal cell carcinoma, *n*	15	10	5	
Squamous cell carcinoma, *n*	2	2	0	
***Melanoma, n (%)***	14 (11.4)	9 (10.7)	5 (13.2)	0.69
**Genitourinary, *n* (%)**	18 (14.8)	14 (16.7)	4 (10.5)	0.54
Wilms tumor, *n*	2	2	0	
Renal oncocytoma, *n*	1	0	1	
Urinary tract carcinoma, *n*	5	3	2	
Renal cell carcinoma, *n*	5	5	0	
Testicular seminoma, *n*	4	3	1	
Gland squamous cell carcinoma, *n*	1	1	0	
**Prostate adenocarcinoma, *n* (%)**	8 (6.6)	5 (6)	3 (7.9)	0.99
**Breast adenocarcinoma, *n* (%)**	15 (12.3)	9 (10.7)	6 (15.8)	0.42
**Thyroid, *n* (%)**	13 (10.7)	10 (11.9)	3 (7.9)	0.72
Papillary carcinoma, *n*	11	10	1	
Follicular carcinoma, *n*	2	0	2	
**Colorectal adenocarcinoma, *n* (%)**	11 (9.0)	6 (7.1)	5 (13.2)	0.28
**Hematopoietic, *n* (%)**	9 (7.4)	7 (8.3)	2 (5.3)	0.82
Non-Hodgkin lymphoma, *n*	4	4	0	
Myeloid acute leukemia, *n*	1	1	0	
Myeloma, *n*	2	2	0	
Essential thrombocythemia, *n*	1	0	1	
Granulocytic dysplasia, *n*	1	0	1	
**Neuroendocrine tumors, *n* (%)**	4 (3.3)	3 (3.6)	1 (2.6)	0.78
**Head–neck cancer, *n* (%)**	3 (2.5)	1 (1.2)	2 (5.3)	0.47
Larynx, *n*	1	0	1	
Salivary glands adenocarcinoma, *n*	1	0	1	
Pharynx squamous cell carcinoma, *n*	1	1	0	
**Liver, *n* (%)**	3 (2.5)	1 (1.2)	2 (5.3)	0.47
Hepatocellular carcinoma, *n*	2	0	2	
Gallbladder adenocarcinoma, *n*	1	1	0	
**Endometrial adenocarcinoma, *n* (%)**	2 (1.6)	2 (2.4)	0	n.a.
**Lung adenocarcinoma, *n* (%)**	1 (0.8)	1 (1.2)	0	n.a.
**Others, *n* (%)**	4 (3.3)	3 (3.6)	1 (2.6)	0.78
Pituitary adenoma, *n*	1	0	1	
Thoracic ganglioneuroma, *n*	1	1	0	
Liposarcoma, *n*	1	1	0	
Low grade sarcoma, *n*	1	1	0	

Abbreviations: IBD = inflammatory bowel disease; CD = Crohn’s Disease: UC = ulcerative colitis; *n* = number; n.a. = not applicable.

**Table 4:** Immunosuppressors, biologics and small molecules after index cancer.

	Immunosuppressors			Biologics					Small Molecules			
IBD patients, *n*=	27			113					9			
	Thiopurines (*n* = 10)	MTX (*n* = 14)	Others (*n* = 3)	TNFi (*n* = 36)	VDZ (*n* = 60)	UST (*n* = 45)	RISA (*n* = 5)	SECUK (*n* = 1)	TOFA (*n* = 3)	FILGO (*n* = 1)	UPA (*n* = 4)	OZA (*n* = 1)
Duration, median [range], months	33.5 [4–200]	31 [16–40]	72 [60–108]	38 [3–180]	24 [3–84]	24 [3–66]	6 [3–12]	48 [48–48]	17 [3–18]	12 [12–12]	6 [5–6]	4 [4–4]
	**Immunosuppressors**			**Biologics**					**Small Molecules**			
**CD Patients, *n*=**	**19**			**76**					**2**			
	**Thiopurines** **(*n* = 10)**	**MTX** **(*n* = 8)**	**Others** **(*n* = 1)**	**TNFi** **(*n* = 29)**	**VDZ** **(*n* = 33)**	**UST** **(*n* = 35)**	**RISA** **(*n* = 5)**	**SECUK** **(*n* = 0)**	**TOFA** **(*n* = 0)**	**FILGO** **(*n* = 0)**	**UPA** **(*n* = 2)**	**OZA** **(*n* = 0)**
Duration, median [range], months	33.5 [4–200]	31 [16–40]	72 [72–72]	39 [3–180]	21 [3–72]	24 [6–66]	6 [3–12]	n.a.	n.a.	n.a.		n.a.
	**Immunosuppressors**			**Biologics**					**Small Molecules**			
**UC Patients, *n*=**	**8**			**37**					**7**			
	**Thiopurines** **(*n* = 0)**	**MTX** **(*n* = 6)**	**Others** **(*n* = 2)**	**TNFi** **(*n* = 7)**	**VDZ** **(*n* = 28)**	**UST** **(*n* = 10)**	**RISA** **(*n* = 0)**	**SECUK** **(*n* = 1)**	**TOFA** **(*n* = 3)**	**FILGO** **(*n* = 1)**	**UPA** **(*n* = 2)**	**OZA** **(*n* = 1)**
Duration, median [range], months	n.a.	15.5 [6–131]	84 [60–108]	29 [5–39]	24 [3–60]	18.5 [3–24]	n.a.	48 [48–48]	17 [3–18]	12 [12–12]	6 [6–6]	4 [4–4]

Abbreviations: MTX = methotrexate; TNFi = tumor necrosis factor-α inhibitor, VDZ = vedolizumab, UST = ustekinumab; Risa = risankizumab; SEKU = secukinumab; TOFA = tofacitinib; FILGO = filgotinib; IPA = upadacitinib; Oza = ozanimod; n.a. = not applicable.

**Table 5:** IBD patients treated with immunomodulators after first cancer subsequently developing new/recurrent cancer.

Pts.	IBD Features	Age at IBD Diagnosis (Years)	Age at Cancer Diagnosis (Years)	Index Cancer	ISSs Before Index Cancer: Type (Duration, mos)	Biologics Before Index Cancer: Type (Duration, mos)	ISS Treatment After Index Cancer, Duration (mos)	Biologic Use After Index Cancer, Duration (mos)	Time Interval from Index Cancer to IMMs (mos)	New Cancer	Recurrent Cancer	Time Interval from Biologics to New/ Recurrent Cancer	Time Interval from New/Recurrent Cancer to Previous Cancer (mos)	Follow-Up Duration After Index Cancer (Years)
1	CD, L3 B2	21	29	Thyroid	n.a.	n.a.	n.a.	ADA (96)	36	n.a.	Thyroid	0	12	10
2	CD, L1, B2	16	17	Melanoma	n.a.	n.a.	n.a.	ADA (9)	6	Melanoma	n.a.	3	3	10
3	CD, L1/L4, B2	22	29	NMSC	AZA (72)	IFX (3)	n.a.	UST (14)	6	NMSC	n.a.	14	168	15
4	CD, L3, B3, *p*	18	57	Prostate NMSC	n.a.	IFX (84) VDZ (12)	n.a.	VDZ (16) UST (42)	6	NMSC	Prostate	16	24	5
5	CD, L3, B1, *p*	46	41	Thyroid	n.a.	n.a.	n.a.	VDZ (53)	132	n.a.	Thyroid	53	183	15
6	UC, E3	55	55	HCC	n.a	n.a.	Tacrolimus/ Everolimus (108)	VDZ (10)	96	Salivary gland carcinoma NMSC	n.a.	96	96	8
7	UC, E3	74	79	Prostate	n.a.	n.a.	n.a.	VDZ (41)	12	n.a.	Prostate	41	53	7
8	UC, E2	25	48	CRC	n.a.	n.a.	MTX (26)	VDZ (9)	24 ISS 60 BIO	n.a.	CRC	10	60	9
9	CD L3 B2	52	57	NMSC	AZA (36)	n.a.	n.a.	ADA (60)	12	NMSC	n.a.	48	60	6
10	CD, L3, B2	29	64	NMSC	AZA (24)	IFX (12) ADA (84)	n.a.	ADA (120)	3	NMSC	n.a.	108	108	3
11	UC, E2	60	59	Renal cell carcinoma	n.a.	n.a.	MTX (108)	VDZ (31) TOFA (17)	144	NMSC	n.a.	36	180	11
12	CD, L2, B1	19	33	Melanoma	AZA (228)	IFX (2) ADA (3)	n.a.	VDZ (3) UST (23)	72	Melanoma NMSC	n.a.	8	60	8

Abbreviations: UC: ulcerative colitis, CD: Crohn’s disease; IBD: inflammatory bowel disease; mos: months; ISSs: immunosuppressors; IMMs: immunomodulators; L1: ileal CD; L2: colonic CD; L3: ileo-colonic CD; B1: non-stricturing–non-penetrating; B2: fibrostricturing; B3: penetrating CD: perianal disease; E2: left-sided colitis; E3: extensive colitis; NMSC: non-melanoma skin cancer; HCC: hepatocellular carcinoma; AZA: azathioprine; IFX: infliximab; ADA: adalimumab VDZ: vedolizumab; UST: ustekinumab; TACR: tacrolimus; EVER: everolimus; MTX: methotrexate; TOFA: tofacitinib; CRC: colorectal cancer; yrs: years; NMSC: non-melanotic skin cancer; HCC: hepatocellular carcinoma; n.a.: not applicable.

**Table 6:** Comparisons in terms of immunomodulatory treatments before index cancer in patients with versus without new or recurrent cancer.

IMMs Before Index Cancer	New/Recurrent Cancer (*n* = 12)	No new/Recurrent Cancer (*n* = 110)	*p*
**ISSs, *n* (%)**	6 (50)	35 (31.8)	0.48
Thiopurines	4 (66.6)	30 (85.7)	0.93
Methotrexate	1 (16.7)	4 (11.4)	0.96
Other	1 (16.7)	3 (8.6)	0.9
**ISS duration, median [range]**			
Thiopurines	54 [24–228]	38 [3–156]	0.11
Methotrexate	3 [3–3]	45.5 [12–89]	0.06
Others	96 [96–96]	19.5 [3–96]	n.a.
**Biologics/JAKi, *n* (%)**	6 (50)	40 (36.4)	0.71
TNFi	6 (100)	34 (85)	0.43
Vedolizumab	1 (16.6)	9 (22.5)	0.64
Ustekinumab	0 (0)	4 (10)	n.a.
Tofacitinib	0 (0)	1 (2.5)	n.a.
**Biologics/JAKi duration median, [range]**			
TNFi	46.5 [3–96]	36 [6–180]	0.85
Vedolizumab	12 [12–12]	6.5 [3–63]	0.55
Ustekinumab	0	19 [1–37]	n.a.
Tofacitinib	0	18 [18–18]	n.a.

Abbreviations: IMMs = immunomodulators; ISSs = conventional immunosuppressors; JAKi = Janus-kinase inhibitor; TNFi = tumor necrosis factor-α inhibitor, n.a. = not applicable.

**Table 7:** Comparisons in terms of immunomodulatory treatments after index cancer between patients with versus without new or recurrent cancer.

	New/Recurrent Malignancy (*n* = 12)	No new/Recurrent Malignancy (*n* = 110)	*p*
**ISSs after cancer, *n* (%)**	4 (30.8)	23 (21.1)	0.65
Thiopurines	1 (25)	9 (39.1)	0.64
Methotrexate	2 (50)	12 (52.3)	0.99
Others	1 (25)	2 (8.7)	0.73
**ISS duration after cancer, median [range]**	25.5 [12–108]	33 [4–200]	0.24
Thiopurines	n.a.	42 [4–200]	n.a.
Methotrexate	n.a.	24.5 [6–131]	n.a.
Others	n.a.	66 [60–72]	n.a.
**Biologics after cancer, *n* (%)**			
TNFi	5 (38.5)	31 (31.3)	0.66
Vedolizumab	8 (61.5)	52 (51.5)	0.73
Ustekinumab	5 (35.5)	40 (40.4)	0.85
Risankizumab	0 (0)	5 (5)	n.a.
Secukinumab	0 (0)	1 (1)	n.a.
**JAKi after cancer, *n* (%)**	1 (7.7)	8 (7.3)	
Tofacitinib	1 (7.7)	2 (2)	n.a.
Filgotinib	0 (0)	1 (1)	n.a.
Upadacitinib	0 (0)	4 (4)	n.a.
Ozanimod	0 (0)	1 (1)	n.a.
**Biologic duration after neoplasia, median [range]**			
TNFi	84 [9–120]	36 [3–180]	0.17
Vedolizumab	13 [3–53]	24 [3–84]	0.36
Ustekinumab	14 [12–42]	24 [3–66]	0.34
Risankizumab	0	6 [3–12]	n.a.
Secukinumab	0	48 [48–48]	n.a.
**JAKi duration after neoplasia, median [range]**			
Tofacitinib	17 [17–17]	17 [3–18]	n.a.
Filgotinib	n.a.	12 [12–12]	n.a.
Upadacitinib	n.a.	6 [5–6]	n.a.
Ozanimod	n.a.	4 [4–4]	n.a.

Abbreviations: ISSs = conventional immunosuppressors; JAKi = Janus-kinase inhibitor; TNFi = tumor necrosis factor-α inhibitor, n.a. = not applicable.

**Table 8:** Univariate analysis: risk factors for new/recurrent cancer.

	OR [CI 95%]	*p*
ISSs after index cancer	1.66 [0.46–5.88]	0.43
ISSs before index cancer	1.81 [0.56–5.79]	0.31
Biologics before index cancer	1.47 [0.46–4.7]	0.51
Age at diagnosis of cancer	0.98 [0.95–1.02]	0.58
Active smoking	0.49 [0.12–1.91]	0.3
Gender (F)	0.45 [0.13–1.55]	0.2
IBD type (CD)	1.57 [0.4–6.1]	0.5
TNFis after index cancer	1.39 [0.42–4.59]	0.58
ISS duration after index cancer	0.6 [0.07–5.07]	0.64
ISS duration before index cancer	1.51 [0.37–6.06]	0.55
TNFi duration after index cancer	3.01 [0.81–11.11]	0.09
TNFi duration before index cancer	1.62 [0.4–6.51]	0.49

Abbreviations: ISSs = conventional immunosuppressors; TNFis = tumor necrosis factor-α inhibitors; CD = Crohn’s Disease; F = Female.

## Data Availability

The data presented in the study are available upon reasonable request.
